# Cost-effectiveness of combining finerenone and sodium-glucose cotransporter 2 inhibitors with standard of care for patients with chronic kidney disease and type 2 diabetes in China 

**DOI:** 10.1080/0886022X.2025.2578413

**Published:** 2025-11-02

**Authors:** Zhengqiang Hu, Wei Jin, Shengying Lou, Lejun Zhang

**Affiliations:** ^a^ Department of Pharmacy, the Fourth Affiliated Hospital of School of Medicine, and International School of Medicine, International Institutes of Medicine, Zhejiang University, Yiwu, China; ^b^ Department of Clinical Research Center, the Fourth Affiliated Hospital of School of Medicine, and International School of Medicine, International Institutes of Medicine, Zhejiang University, Yiwu, China

**Keywords:** Cost-effectiveness analysis, chronic kidney disease, type 2 diabetes, finerenone, sodium-glucose cotransporter 2 inhibitors

## Abstract

**Objective:**

To evaluate the cost-effectiveness of combining finerenone and Sodium-glucose cotransporter-2 inhibitors (SGLT2i) with standard care (SoC) for Chinese patients with chronic kidney disease (CKD) and type 2 diabetes (T2D).

**Methods:**

A validated Markov model (*FINE-CKD*) was employed to simulate health outcomes and costs from a Chinese healthcare system perspective. Transition probabilities, costs, and utilities were derived from peer-reviewed literature and trial data. The model utilized a 4-month cycle length, consistent with the assessment intervals in the FIDELITY trial, over a ten-year time horizon. The primary economic outcome was the net monetary benefit (NMB). Cost-effectiveness was assessed against China’s willingness-to-pay (WTP) threshold, defined as three times the 2023 GDP per capita (268,074 CNY/QALY), following China’s pharmacoeconomic evaluation guidelines.

**Results:**

Triple therapy (finerenone + SGLT2i + SoC) demonstrated superior clinical and economic outcomes compared to finerenone therapy (finerenone + SoC) and SGLT2i therapy (SGLT2i + SoC). Specifically, compared to finerenone therapy, triple therapy resulted in cost savings of CNY 102,953.11 and an incremental gain of 0.291 quality-adjusted life years (QALYs), yielding an NMB of CNY 181,032.95. Compared to SGLT2i therapy, triple therapy led to cost savings of CNY 118,628.19 and an additional 0.257 QALYs, corresponding to an NMB of CNY 187,506.53. Sensitivity analyses confirmed the robustness of these findings.

**Conclusions:**

Triple therapy with finerenone, SGLT2i, and SoC is cost-effective for Chinese patients with T2D and CKD, delivering synergistic cardiovascular and renal protective effects while demonstrating robust economic viability within China’s healthcare framework.

## Graphical Abstract

**Figure:**
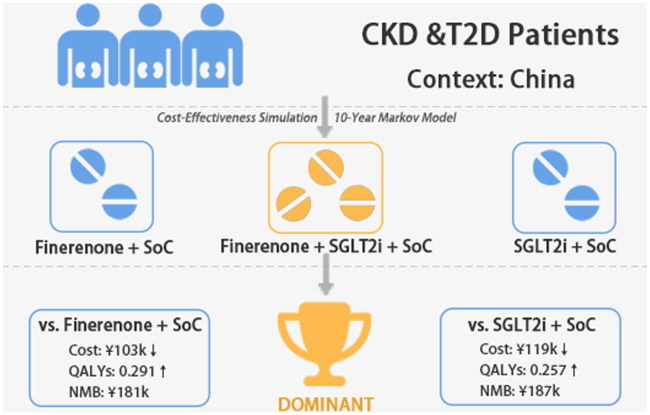

## Introduction

Diabetic kidney disease (DKD), a severe and progressive microvascular complication of diabetes mellitus, is widely recognized as one of the primary contributors to end-stage renal disease (ESRD) globally. DKD not only accelerates renal function decline but also significantly increases cardiovascular risks. Its high prevalence and disability burden severely impair patients’ quality of life while imposing substantial socioeconomic costs. The rising prevalence of DKD poses significant challenges to healthcare systems, particularly in low- and middle-income countries where access to advanced treatments remains limited due to financial constraints and fragmented healthcare infrastructure. According to the International Diabetes Federation (IDF), approximately 537 million individuals globally were diagnosed with diabetes in 2021, with projections estimating a rise to 783 million by 2045 [1]. Diabetes is a primary contributor to chronic kidney disease (CKD), with up to 40% of diabetic patients progressing to CKD [2], a trajectory intensified by delayed diagnosis and suboptimal glycemic control in low-resource regions. Global epidemiological studies reveal a staggering 94% increase in DKD-attributable mortality between 1990 and 2012 [3]. In China, rapid urbanization and lifestyle transitions have significantly increased the diabetes burden, with prevalence rates rising from 10.9% in 2013 to 12.4% during 2018–2019 [4]. Notably, T2D constitutes over 90% of diagnosed cases [4]. Emerging challenges are amplified by marked urban-rural disparities in healthcare accessibility, compounded by aging populations and escalating obesity rates. The synergistic progression of T2D and CKD has emerged as a critical public health challenge, posing significant threats to both individual health outcomes and healthcare system sustainability. This dual burden demands interventions that optimize both clinical efficacy and cost-effectiveness, particularly in resource-limited settings where budgetary constraints amplify the need for economically viable solutions. Particularly concerning is the substantial economic burden imposed by advanced CKD management, which not only strains healthcare budgets but also limits patient access to optimal care, highlighting the imperative for implementing preventive strategies to mitigate disease progression through cost-conscious therapeutic approaches.

Renin-angiotensin system inhibitors (RASi), including angiotensin-converting enzyme inhibitors (ACEi) and angiotensin receptor blockers (ARBs), have long been established as first-line agents for the standard of care (SoC) in DKD management [5–7]. However, significant residual risks of CKD progression and cardiovascular (CV) events persist despite optimized RASi therapy [3], compounded by adverse effects such as hyperkalemia that limit their use in vulnerable populations [8]. Novel agents—including SGLT2i and ns-MRAs such as finerenone—exhibit complementary mechanisms to address unmet clinical needs [9]. SGLT2i demonstrate robust reductions in cardiovascular events, CKD progression, and all-cause mortality among patients with T2D [10], while finerenone mitigates cardiovascular morbidity and renal function decline in T2D patients with albuminuria [11]. Mechanistic distinctions between these drug classes—targeting tubular glucose reabsorption (SGLT2i) and mineralocorticoid receptor overactivation (ns-MRAs)—support their combined use, with emerging evidence from preclinical and clinical studies suggesting amplified clinical benefits when co-administered [12–15]. For instance, preclinical models demonstrate that dual inhibition of SGLT2 and mineralocorticoid receptors synergistically reduces fibrosis and inflammation across renal compartments [16]. These findings align with clinical observations from the FIDELITY analysis—a pooled dataset of two pivotal phase III trials (FIDELIO-DKD and FIGARO-DKD) evaluating finerenone in over 13,000 patients—where finerenone exhibited consistent cardiorenal benefits irrespective of SGLT2i use [11,17–19]. Notably, concomitant SGLT2i use may attenuate finerenone-associated hyperkalemia without increasing the risk of renal injury [19–22].

Despite these advances, the economic implications of combination therapy remain underexplored, particularly in resource-constrained settings like China, where rising healthcare expenditures necessitate cost-effective strategies. Previous studies support the cost-effectiveness of adding either SGLT2i or finerenone to SoC [23,24]^,^, but none have evaluated their combined use. This study aims to bridge this gap by assessing the cost-effectiveness of finerenone plus SGLT2i combination therapy versus monotherapy in Chinese patients with T2D-associated CKD, leveraging clinical data from the FIDELITY subanalysis [19]. The findings will inform clinical guidelines and reimbursement policies.

## Methods

### Study design

We conducted a cost-effectiveness analysis from the perspective of the Chinese healthcare system, comparing three treatment strategies for patients with CKD and T2D: Finerenone + SGLT2i + SoC (triple therapy), SGLT2i + SoC (SGLT2i therapy), and Finerenone + SoC (finerenone therapy). The dosing regimens were selected based on the maximum maintenance doses specific to each drug class, in accordance with Chinese prescribing guidelines and clinical trial protocols.

### Study population

The baseline characteristics of the study cohort were consistent with the FIDELITY trial population [19]. Specifically, a total of 13,026 patients were included. Of these, 877 (6.7%) received an SGLT2i at baseline, comprising 438 (6.7%) of 6,519 in the finerenone group and 439 (6.7%) of 6,507 in the placebo group. The median follow-up period for the FIDELITY analysis was 3.0 years (interquartile range 2.3–3.8 years). Patients were stratified into CKD stages 1/2, 3, and 4 according to estimated glomerular filtration rate (eGFR) values at baseline, as shown in Tables S1, S2 of Supplemental data.

**Table 1.** Transition probabilities matrix of SoC.

To From	CKD 1/2 (%)	CKD 3 (%)	CKD 4 (%)	CKD 5 without RRT (%)	Dialysis (acute) (%)	Dialysis (post-acute) (%)	Kidney transplant (acute) (%)	Kidney transplant (post-acute) (%)	Source
CKD 1/2	59.38	40.38	0.24	0	0	0	0	0	[23]
CKD 3	2.85	87.35	9.51	0.09	0.13	0	0.07	0	
CKD 4	0	10.92	81.24	5.22	2.62	0	0	0	
CKD 5 without RRT	0	0.39	4.25	43.7	49.67	0	1.99	0	
Dialysis (acute)	0	0	0	0	0	100	0	0	
Dialysis (post-acute)	0	0	0	0	0	98.73	1.27	0	
Kidney transplant (acute)	0	0	0	0	0	0	0	100	
Kidney transplant (post-acute)	0	0	0	0	0	0	0	100	

SoC, standard of care; CKD, chronic kidney disease.

**Table 2.** Clinical Inputs.

	Base case	Range	Distribution	Source
First CV event risk per cycle, SoC				[23]
CKD 1/2	1.26%	0.41% − 2.56%	Beta	
CKD 3	0.95%	0.69% − 1.26%	Beta	
CKD 4	1.71%	1.12% − 2.42%	Beta	
CKD 5 without RRT	2.24%	0.47% − 5.33%	Beta	
Dialysis (acute)	2.24%	0.47% − 5.33%	Beta	
Dialysis (post-acute)	2.24%	0.47% − 5.33%	Beta	
Kidney transplant (acute)	1.71%	1.12% − 2.42%	Beta	
Kidney transplant (post-acute)	1.71%	1.12% − 2.42%	Beta	
Subsequent CV event risk, SoC	8.50%	5.97% − 11.42%	Beta	[19]
HR: First CV event risk, Finerenone therapy vs SoC	0.87	0.70 − 1.04	Lognormal	
HR: First CV event risk, SGLT2i therapy vs SoC	0.80	0.64 − 0.96	Lognormal	
HR: First CV event risk, triple therapy vs SoC	0.57	0.46 − 0.69	Lognormal	
All cause death risk per cycle, SoC				
CKD 1/2	0.92%	0.60% − 1.66%	Beta	[23]
CKD 3	0.58%	0.45% − 0.76%	Beta	
CKD 4	0.85%	0.55% − 1.27%	Beta	
CKD 5 without RRT	1.60%	0.34% − 5.06%	Beta	
Dialysis	5.01%	0.57% − 6.34%	Beta	
Kidney transplant	0.85%	0.55% − 1.27%	Beta	
HR: All-cause mortality probability, Finerenone therapy vs SoC	0.90	0.72 − 1.08	Lognormal	[19]
HR: All-cause mortality probability, SGLT2i therapy vs SoC	0.70	0.56 − 0.84	Lognormal	
HR: All-cause mortality probability, triple therapy vs SoC	0.46	0.37 − 0.55	Lognormal	
Adverse event risks per cycle				
Finerenone therapy				[19]
Hyperkalemia leading to hospitalization	0.16%	0.12% − 0.20%	Beta	
Acute Kidney Injury	0.40%	0.31% − 0.51%	Beta	
Any AE leading to discontinuation	0.75%	0.59% − 0.97%	Beta	
SGLT2i therapy				
Hyperkalemia leading to hospitalization	0.00%	–	–	
Acute Kidney Injury	0.38%	0.30% − 0.50%	Beta	
Any AE leading to discontinuation	0.59%	0.47% − 0.77%	Beta	
Triple therapy				
Hyperkalemia leading to hospitalization	0.03%	0.02% − 0.03%	Beta	
Acute Kidney Injury	0.12%	0.09% − 0.16%	Beta	
Any AE leading to discontinuation	0.47%	0.37% − 0.61%	Beta	
Treatment efficacy				
Relative eGFR Decline Rate, Finerenone therapy vs SoC	0.68	0.55 − 0.82	Lognormal	[19]
Relative eGFR Decline Rate, SGLT2i therapy vs SoC	0.93	0.74 − 1.11	Lognormal	
Relative eGFR Decline Rate, triple therapy vs SoC	0.52	0.41 − 0.62	Lognormal	
HR: Renal Composite Outcome, Finerenone therapy vs SoC	0.78	0.62 − 0.94	Lognormal	
HR: Renal Composite Outcome, SGLT2i therapy vs SoC	0.52	0.42 − 0.62	Lognormal	
HR: Renal Composite Outcome, triple therapy vs SoC	0.27	0.21 − 0.32	Lognormal	

SoC, standard of care; CKD, chronic kidney disease; RRT, renal replacement therapy; HR, hazard ratio; SGLT2i, sodium-glucose cotransporter 2 inhibitors; AE, Adverse event; eGFR, estimated glomerular filtration rate.

### Model structure

**Figure 1.**
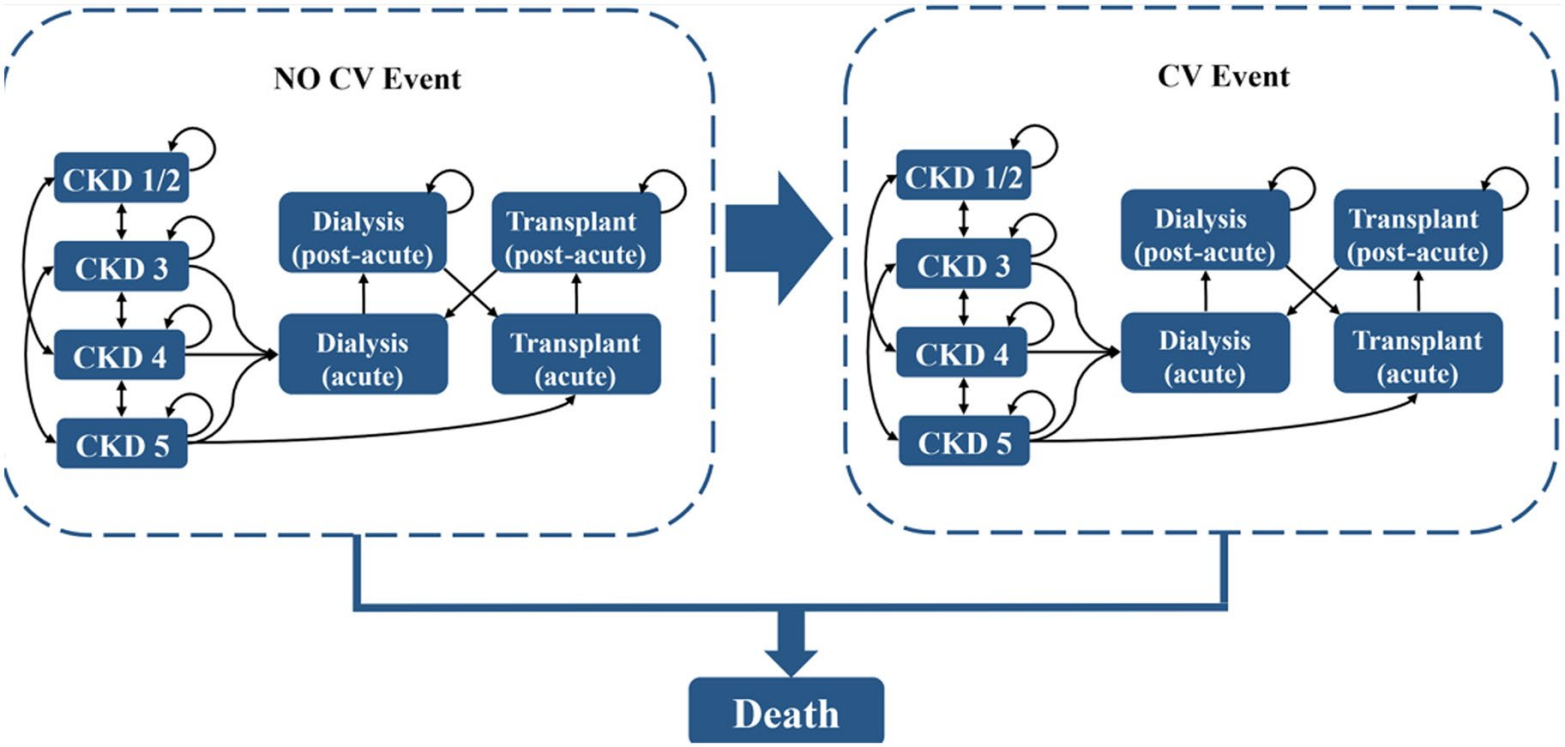
Schematic diagram of the chronic kidney disease Markov model. CV, cardiovascular; CKD, chronic kidney disease.

In this study, a validated Markov model (*FINE-CKD*) was utilized to simulate health outcomes and associated costs from the perspective of the Chinese healthcare system [25,26]. The model employed four-month cycles over a ten-year time horizon, aligning with the chronic nature of CKD and T2D. Health states were defined by CKD stages (1/2, 3, 4, 5 without RRT, dialysis, transplant) and cardiovascular (CV) event history (Figure 1). The model explicitly differentiated between acute and post-acute phases of dialysis and kidney transplantation to capture short-term cost spikes and long-term maintenance expenses. For instance, acute dialysis was assigned higher immediate costs due to hospitalization, while post-acute dialysis reflected ongoing outpatient expenditures. Similarly, kidney transplant costs were bifurcated into initial surgical expenses and subsequent immunosuppressive therapy costs. Patients transitioned between states based on predefined probabilities, with death as an absorbing state. First CV events triggered irreversible transitions to corresponding post-CV health states, reflecting the irreversible impact of cardiovascular morbidity on long-term outcomes. Adverse events (AEs)—hyperkalemia leading to hospitalization and acute kidney injury (AKI)—were modeled as disutility and cost modifiers without affecting disease progression.

### Transition probabilities

The baseline transition probability matrix for the SoC arm was primarily informed by published literature [23]. For triple therapy, SGLT2i therapy, and finerenone therapy, their respective transition probabilities were derived by adjusting the SoC probabilities based on relative treatment effects reported in the FIDELITY subanalyses [19]. Specifically, adjustments for transitions among CKD stages 1–4 were based on the differences in chronic eGFR slope for each intervention relative to SoC, while adjustments for the probabilities of late-stage renal events post-CKD stage 4 (including CKD5 without RRT dialysis, and kidney transplantation) were based on the HR for composite kidney outcomes for each intervention relative to SoC. The transition probability matrices of SoC are detailed in Table 1.

### Event risks and mortality

Key clinical events incorporated into the model include CV events, AEs, and all-cause mortality.

#### CV Events

CV events encompassed non-fatal myocardial infarction, non-fatal ischemic stroke, non-fatal hemorrhagic stroke, and hospitalization for heart failure. These individual events were modeled as a composite CV event, weighted by their respective incidence proportions within the target population (Table S3 of supplemental data). The model differentiated between initial and subsequent CV events. The incidence rates for both initial and subsequent CV events under SoC were sourced from published literature^[^^23^^]^. For triple therapy, SGLT2i therapy, and finerenone therapy, their corresponding CV event rates were calculated by adjusting the SoC rates using strategy-specific HRs relative to SoC, as derived from the FIDELITY subanalysis [19].

#### AEs

The primary AEs considered were hyperkalemia leading to hospitalization and AKI. Annual probabilities for these AEs were obtained from the FIDELITY subanalysis [19] and subsequently converted into 4-month cycle-specific rates using the formula r = -ln(1-P)/t, where P is the annual probability and t is the cycle duration (i.e., 3 cycles per year).

#### All-cause mortality

CKD stage-specific all-cause mortality rates for the SoC arm were derived from published literature [23]. Similar to the CV Events, for the three intervention strategies, their all-cause mortality rates were calculated by adjusting the SoC stage-specific rates using strategy-specific HRs for all-cause mortality relative to SoC, also sourced from the FIDELITY subgroup analysis [19].

All event risk and mortality parameters are detailed in Table 2.

### Cost inputs

The analysis adopted the perspective of the Chinese healthcare system, incorporating direct medical costs. All costs were adjusted to 2023 prices using the Chinese healthcare price index and discounted at a rate of 5%. Cost parameters and sources are detailed in Table 3.

**Table 3.** Cost Inputs.

Health states/events	Base case (CNY)	Range (CNY)	Distribution	References
CKD 1/2, per cycle	269	215–323	Gamma	[23]
CKD 3, per cycle	269	215–323	Gamma	
CKD 4, per cycle	269	215–323	Gamma	
CKD 5 without RRT, per cycle	1800	1440 − 2160	Gamma	
Dialysis (acute), per cycle	54604	43683–65525	Gamma	
Dialysis (post-acute), per cycle	50106	40085–60128	Gamma	
Kidney transplant (acute)	228571	182857–274286	Gamma	
Kidney transplant (post-acute), per cycle	44337	35470–53025	Gamma	
MI (acute)	31267	25014–37520	Gamma	
MI (post-acute), per cycle	12068	9654–14482	Gamma	
Ischemic stroke (acute)	10181	8145–12217	Gamma	
Ischemic stroke (post-acute), per cycle	10567	8453–12680	Gamma	
Hemorrhagic stroke (acute)	21355	17084–25626	Gamma	
Hemorrhagic stroke (post-acute), per cycle	7455	5964 − 8946	Gamma	
Hospitalization for HF (acute)	48429	38743–58115	Gamma	
Hospitalization for HF (post-acute)	6177	4942 − 7413	Gamma	
Hyperkalemia leading to hospitalization	1000	800–1200	Gamma	
Acute Kidney Injury	40445	18727–95320	Gamma	[27]
SoC, per cycle	1561	1249.024 - 1874	Gamma	[23]
Finerenone, per cycle	1445	1156 − 1733	Gamma	[33]
SGLT2i, per cycle	494	285–703	Gamma	
Death	36487	29189–43784	Gamma	[23]

CKD, chronic kidney disease; RRT, renal replacement therapy; HF, heart failure; MI, myocardial infarction; SGLT2i, sodium-glucose cotransporter 2 inhibitors.

#### Drug costs

All drugs included in the treatment strategies were costed based on their maximum recommended maintenance doses in Chinese clinical practice. The costs of drugs included in SoC were obtained from published literature [23]. Prices for finerenone and SGLT2i were sourced from ‘Quanyaowang,’ a market-oriented centralized procurement, supply, and service platform for pharmaceuticals used by public medical institutions in China. The price for SGLT2i represented an average of commercially available drugs within this class.

#### Disease management and treatment costs

Costs associated with the management of different CKD stages, dialysis (both acute and post-acute phases), and kidney transplantation (both acute and long-term post-transplant management) were derived from a published pharmacoeconomic analysis conducted within the Chinese healthcare system [23].

#### CV event costs

CV events included non-fatal myocardial infarction, non-fatal ischemic stroke, non-fatal hemorrhagic stroke, and hospitalization for heart failure. The costs for initial CV events were differentiated into acute phase costs and post-acute management costs, sourced from the same pharmacoeconomic literature as disease management costs [23]. These were subsequently weighted according to the distribution of specific CV event types reported in the FIDELITY subanalysis^[^^19^^]^ (Table S3) to derive the average cost for a composite CV event in the model.

#### AE costs

The cost of managing hyperkalemia leading to hospitalization was sourced from the aforementioned pharmacoeconomic literature [23]. The treatment cost for AKI was obtained from a nationwide, cross-sectional survey conducted in China [27].

### Utility inputs

Health-related quality of life was evaluated based on EQ-5D-5L questionnaire responses obtained from the Asian subpopulation participating in the FIDELIO-DKD trial [23]. These utility values were discounted at an annual rate of 5% and are detailed in Table 4.

**Table 4.** Utility Inputs.

Health states/events	Base case	Range	Distribution	References
CKD 1/2 utility	0.884	0.707 − 1.000	Beta	[23]
CKD 3 utility	0.884	0.707 − 1.000	Beta	
CKD 4 utility	0.884	0.707 − 1.000	Beta	
CKD 5 without RRT utility	0.871	0.696 − 1.000	Beta	
Dialysis (acute) utility	0.804	0.643 − 0.964	Beta	
Dialysis (post-acute) utility	0.782	0.625 − 0.938	Beta	
Kidney transplant (acute) utility	0.813	0.650 − 0.975	Beta	
Kidney transplant (post-acute) utility	0.972	0.777 − 1.000	Beta	
Disutility due to MI (acute)	−0.075	−0.090 to −0.060	Beta	
Disutility due to MI (post-acute)	−0.009	−0.011 to −0.007	Beta	
Disutility due to stroke (acute)	−0.059	−0.071 to −0.047	Beta	
Disutility due to stroke (post-acute)	−0.018	−0.022 to −0.014	Beta	
Disutility due to hospitalization for HF (acute)	−0.012	−0.014 to −0.010	Beta	
Disutility due to hospitalization for HF (post-acute)	−0.07	−0.084 to −0.056	Beta	

CKD, chronic kidney disease; RRT, renal replacement therapy; HF, heart failure; MI, myocardial infarction.

### Statistical and sensitivity analysis

The primary outcomes of the model were cost, QALYs, and NMB. The NMB was calculated using the formula: NMB = (Incremental QALYs × WTP) – Incremental Costs. A positive NMB indicates that the intervention is considered cost-effective compared to the alternative. One-way sensitivity analysis identified key drivers of cost-effectiveness by varying parameters within plausible ranges. Probabilistic sensitivity analysis (PSA) employed Monte Carlo simulations (1,000 iterations) with predefined distributions to estimate the probability of triple therapy being cost-effective across uncertainty ranges.

### Ethical considerations

As a simulation-based study using aggregated data, all input data were anonymized and sourced from publicly available literature.

## Results

### Base-Case analysis

Over a ten-year simulation horizon, the triple therapy was a dominant strategy compared to both finerenone therapy and SGLT2i therapy (Table 5). Specifically, when compared with SGLT2i therapy, triple therapy resulted in cost savings of CNY 118,628.19 and an incremental gain of 0.257 QALYs, yielding a NMB of CNY 187,506.53. Similarly, triple therapy demonstrated substantial economic advantages over finerenone therapy, with cost savings of CNY 102,953.11 and an additional 0.291 QALYs gained, corresponding to an NMB of CNY 181,032.95. These findings suggest that, at the defined willingness-to-pay threshold, triple therapy represents the most economically favorable option among the evaluated strategies.

**Table 5.** Cost-effectiveness results.

	Cost (CNY)	incrCost (CNY)	QALYs	incrQALYs	NMB (CNY)
**SGLT2i therapy**	352,320.33	0.00	4.637	0	0.00
**Triple therapy**	233,692.14	−118,628.19	4.894	0.257	187,506.53
	**Cost (CNY)**	**incrCost (CNY)**	**QALYs**	**incrQALYs**	**NMB (CNY)**
Finerenone therapy	336,645.25	0.00	4.603	0	0.00
Triple therapy	233,692.14	−102,953.11	4.894	0.291	181,032.95

SGLT2i, sodium-glucose cotransporter 2 inhibitors; QALYs, quality-adjusted life years; NMB, net monetary benefit.

### Sensitivity analyses

One-way sensitivity analysis and probabilistic sensitivity analysis were conducted to evaluate the robustness of the model.

**Figure 2.**
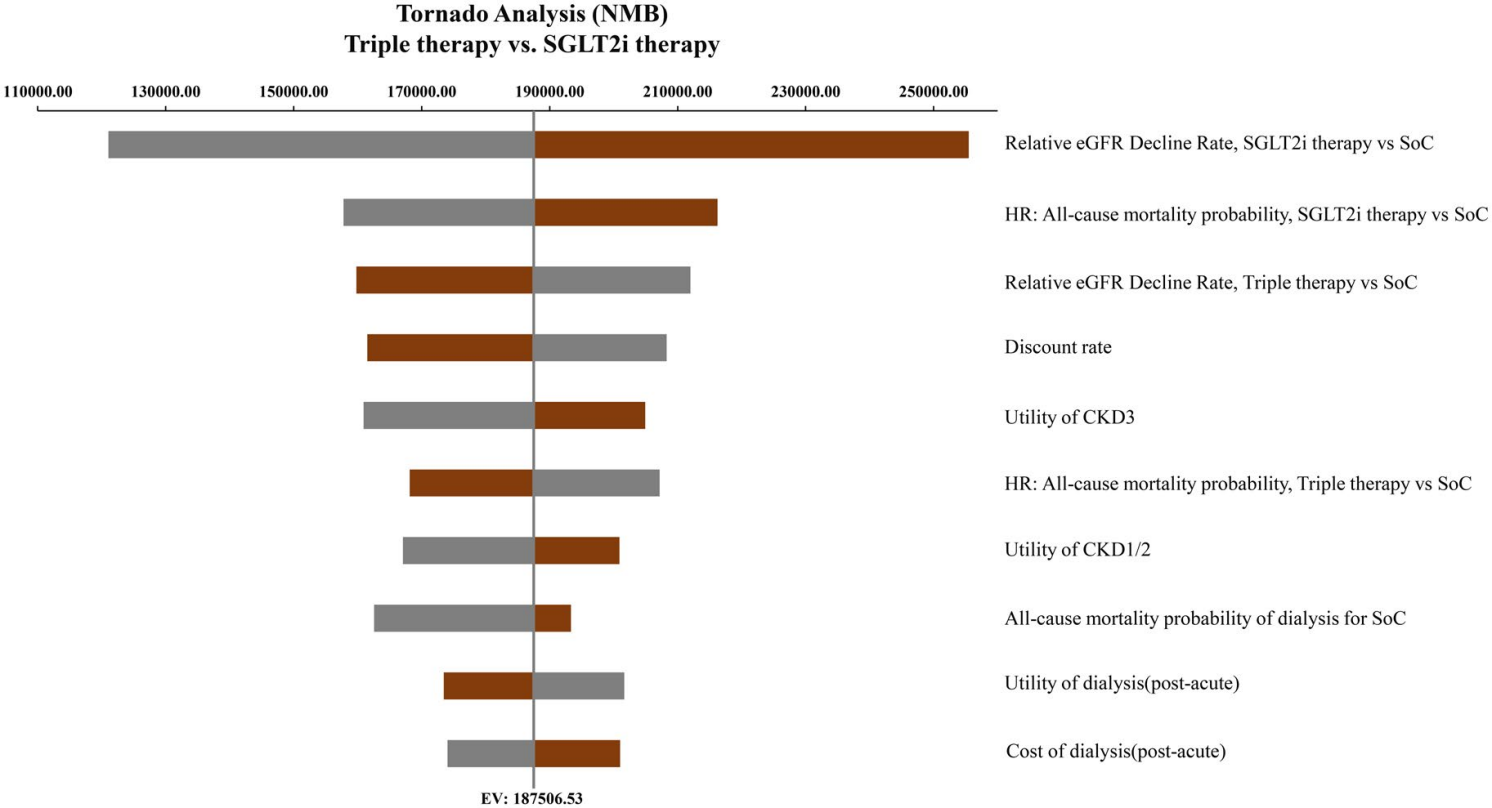
Tornado Analysis of triple therapy vs. SGLT2i therapy. Gray bars: parameter range from lower bound to baseline. Red bars: parameter range from baseline to upper bound. NMB, net monetary benefit; SGLT2i, sodium-glucose cotransporter 2 inhibitors; eGFR, estimated glomerular filtration rate; HR, hazard ratio; SoC, standard of care; CKD, chronic kidney disease. EV: expected value.

**Figure 3.**
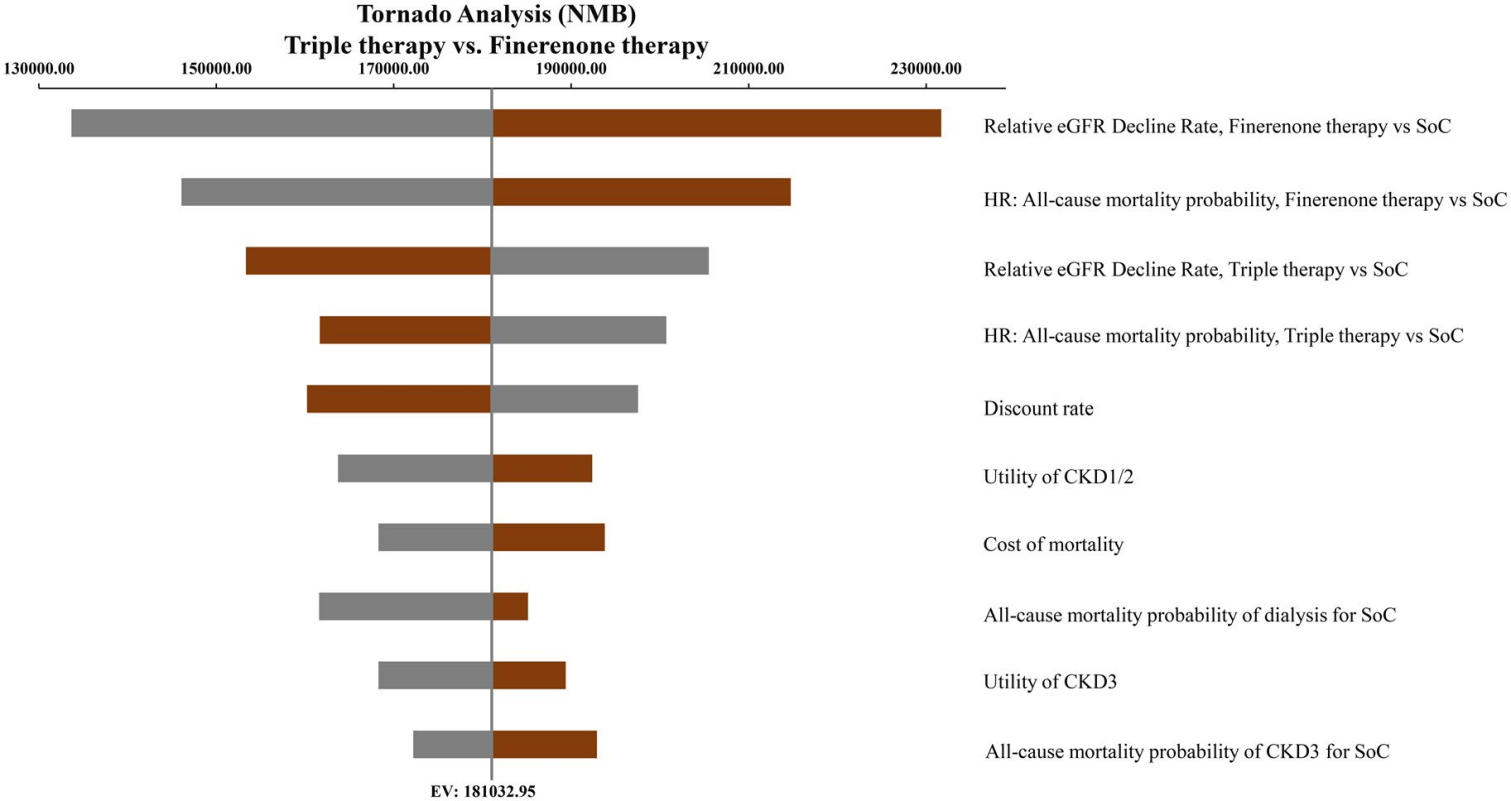
Tornado Analysis of triple therapy vs. Finerenone therapy. Gray bars: parameter range from lower bound to baseline. Red bars: parameter range from baseline to upper bound. NMB, net monetary benefit; eGFR, estimated glomerular filtration rate; HR, hazard ratio; SoC, standard of care; CKD, chronic kidney disease. EV: expected value.

The tornado diagram (Figures 2,3) revealed that when comparing the triple therapy to SGLT2i therapy, the top three parameters with the greatest impact on the NMB were: the relative eGFR decline rate for SGLT2i therapy versus SoC, the HR for all-cause mortality for SGLT2i therapy versus SoC, and the relative eGFR decline rate for triple therapy versus SoC When comparing the triple therapy to finerenone therapy, the relative eGFR decline rate for finerenone therapy versus SoC, the HR for all-cause mortality for finerenone therapy versus SoC, and the relative eGFR decline rate for triple therapy versus SoC were the most impactful parameters.

**Figure 4.**
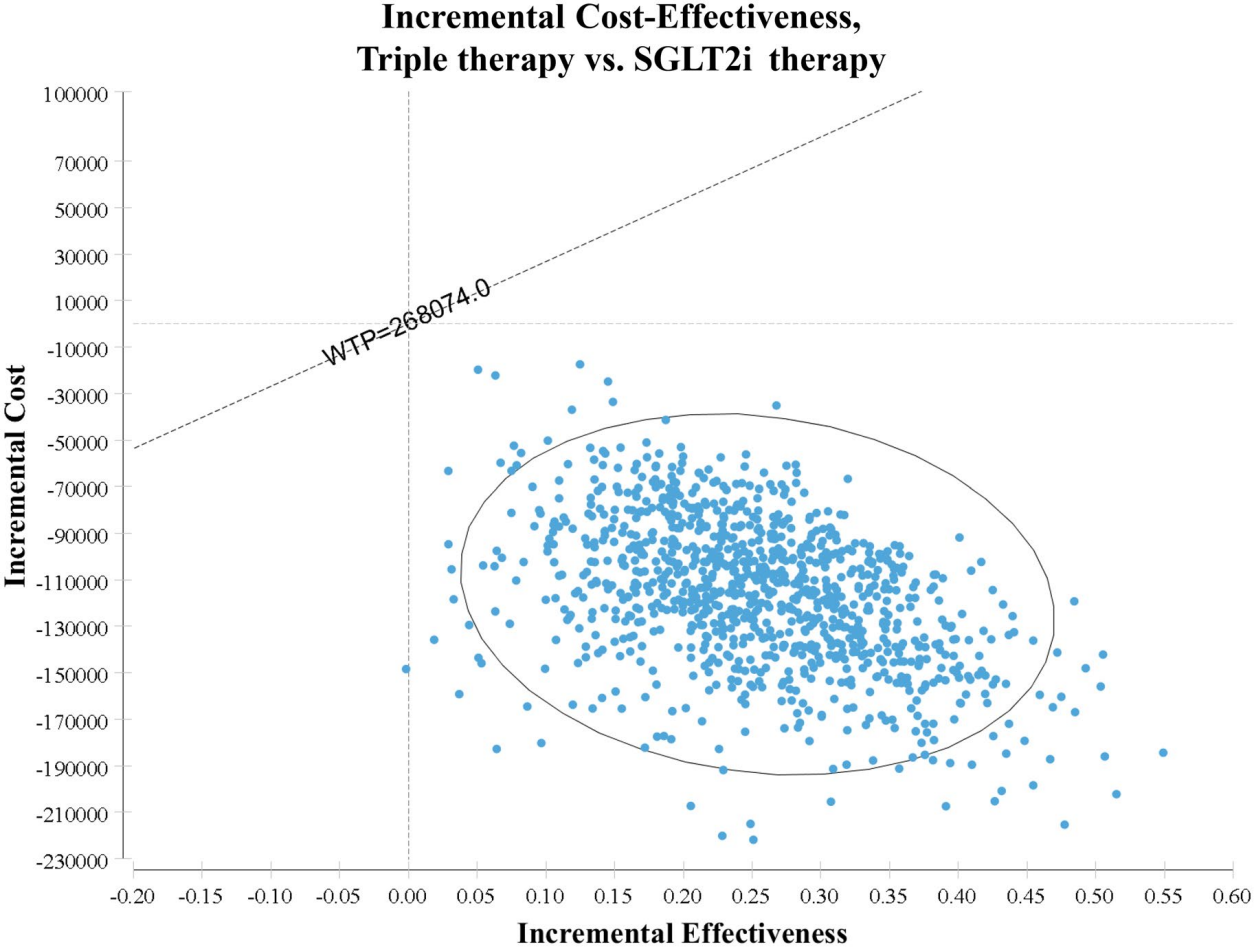
The Incremental Cost-Effectiveness scatter plot of 1000 simulations, triple therapy vs. SGLT2i therapy. SGLT2i, sodium-glucose cotransporter 2 inhibitors; SoC, standard of care; WTP, willingness to pay.

**Figure 5.**
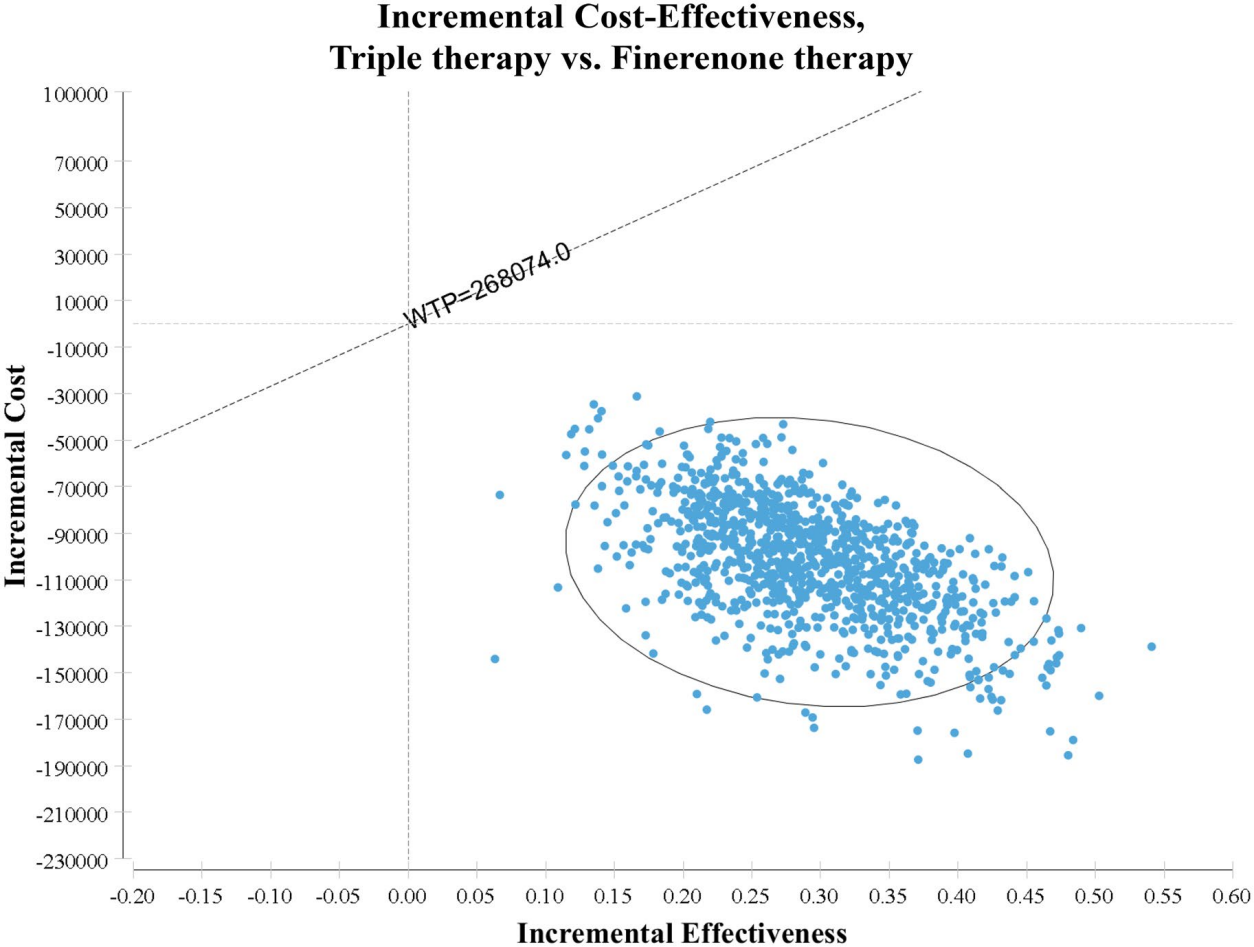
The Incremental Cost-Effectiveness scatter plot of 1000 simulations, triple therapy vs. finerenone therapy. SoC, standard of care; WTP, willingness to pay.

Monte Carlo simulations (1,000 iterations) (Figures 4,5) demonstrated that triple therapy consistently remained the dominant treatment strategy—being both less costly and more effective—compared to SGLT2i therapy and finerenone therapy across the entire range of considered parameter values.

### Scenario analysis

To supplement the base-case analysis (ten-year horizon) and to evaluate the economic performance of triple therapy over a shorter period, a scenario analysis was conducted. In this analysis, the model’s time horizon was set to four years, aligning with the maximum follow-up duration of the FIDELITY trial. The results indicated that even within this shorter four-year simulation, triple therapy remained the dominant strategy compared to both SGLT2i therapy and finerenone therapy (Table S4 of supplemental data). Specifically, the Net Monetary Benefit (NMB) for triple therapy was CNY 17,117.14 when compared to SGLT2i therapy, and CNY 28,434.07 when compared to finerenone therapy. This finding suggests that the economic advantages of triple therapy are not only projected over the long term but are also significant in the short term, thereby providing stronger evidence to support the early initiation of this combination regimen.

## Discussion

Despite the well-established role of RASi as the foundational therapy for managing CKD in patients with T2D, significant residual risks of disease progression and CV complications remain unaddressed under the current SoC. Recent large-scale clinical trials have validated the cardiorenal benefits of finerenone and SGLT2i [17,18,28–30], positioning these agents as guideline-recommended additions to SoC. Mechanistic synergy between finerenone and SGLT2i, supported by preclinical and clinical evidence, suggests enhanced renoprotection and CV risk reduction when used concomitantly [16,19]. While previous cost-effectiveness analyses have demonstrated favorable profiles for both finerenone and SGLT2i as monotherapies adding to SoC [23,24,31,32], this study is the first to evaluate the economic implications of their combination therapy in China.

The escalating global burden of DKD demands therapeutic strategies that not only improve clinical outcomes but also align with economic realities, particularly in resource-constrained settings like China. This study demonstrates that triple therapy—combining finerenone, SGLT2i, and SoC—is a cost-effective approach for managing T2D-associated CKD, offering both clinical and economic advantages over monotherapy regimens. These findings hold critical implications for clinical practice, healthcare policy, and future research in the context of China’s rapidly evolving healthcare landscape.

The superiority of triple therapy stems from the complementary mechanisms of SGLT2i and finerenone, which target distinct pathways in DKD progression. SGLT2i primarily inhibit glucose reabsorption in the proximal tubule, thereby reducing hyperglycemia and intraglomerular pressure, while finerenone blocks mineralocorticoid receptor overactivation, mitigating inflammation, fibrosis, and oxidative stress in podocytes and tubular cells [9]. Preclinical studies in Alport syndrome models have shown that dual SGLT2 and mineralocorticoid receptor inhibition synergistically attenuates glomerulosclerosis and tubulointerstitial fibrosis, preserving renal architecture more effectively than either agent alone [16]. Clinically, the FIDELITY subanalysis revealed that finerenone’s cardiorenal benefits were consistent irrespective of SGLT2i use, suggesting additive rather than competitive effects [19]. Furthermore, SGLT2i may counteract finerenone-associated hyperkalemia by enhancing urinary potassium excretion, a safety advantage observed in pooled trial data [19–21]. This mechanistic synergy not only enhances efficacy but also reduces adverse events, making the combination both clinically viable and economically favorable.

Our findings robustly demonstrate that triple therapy is a dominant strategy compared to both finerenone therapy and SGLT2i therapy in patients with T2D and CKD in China over a ten-year horizon. Specifically, triple therapy yields substantial cost savings (CNY 102,953.11 and CNY 118,628.19, respectively) and notable improvements in health outcomes (0.291 and 0.257 additional QALYs, respectively), resulting in significantly positive Net Monetary Benefits (CNY 181,032.95 and CNY 187,506.53, respectively) against both comparators at China’s willingness-to-pay threshold. One-way sensitivity analysis further reveals that this pronounced advantage is primarily attributable to triple therapy’s superior efficacy in delaying CKD progression—as reflected by its impact on the annual rate of eGFR decline—and its reduction in all-cause mortality. Furthermore, the economic advantage of triple therapy is amplified in China’s context, where lower drug acquisition costs for generics—driven by national volume-based procurement policies—and streamlined reimbursement mechanisms enhance affordability. Compared to Western healthcare systems, where high drug prices might offset combination therapy benefits, China’s cost structure allows greater alignment between clinical efficacy and economic feasibility.

Despite the robust findings, this study has several limitations. Firstly, the model’s parameters were primarily informed by the FIDELITY trial and its subgroup analyses, wherein the number of patients receiving SGLT2 inhibitors at baseline was relatively small, an imbalance that may introduce uncertainty into the parameter estimates. Secondly, the adjustment factors used to derive strategy-specific CKD state transition probabilities—calculated from differences in annual eGFR decline rates or hazard ratios for composite kidney outcomes—represented an average effect over the entire follow-up period of the FIDELITY trial, potentially masking dynamic changes in treatment efficacy over time. Thirdly, our model extrapolated short-term clinical trial data over a ten-year horizon, which introduces uncertainty regarding the long-term durability of treatment effects. Therefore, while our analysis suggests substantial long-term health-economic benefits from the early initiation of triple therapy, these limitations underscore that regular reassessment of a patient’s clinical status, treatment response, and risk-benefit profile remains crucial in clinical practice. Future long-term follow-up data and real-world evidence are essential for validating our model’s assumptions and refining long-term treatment strategies.

## Conclusions

From a Chinese healthcare system perspective, triple therapy with finerenone, SGLT2i and SoC demonstrates superior cost-effectiveness compared to either finerenone or SGLT2i as add-ons to SoC in patients with T2D and CKD. These findings provide robust economic evidence supporting the integration of dual cardiorenal protection (finerenone + SGLT2i) into clinical practice, with the potential to optimize resource allocation and improve long-term patient outcomes. As the global diabetes epidemic escalates, China’s experience may offer valuable lessons for other middle-income countries grappling with similar dual burdens of disease and resource constraints.

## Supplementary Material

supplemental data.docx
