# Supplementary material for: Cost-effectiveness of combining finerenone and sodium-glucose cotransporter 2 inhibitors with standard of care for patients with chronic kidney disease and type 2 diabetes in China
Source: Ren Fail. 2025 Nov 2;47(1):2578413. doi: 10.1080/0886022X.2025.2578413 (PMC12584830; doi:10.1080/0886022X.2025.2578413)
Supplement: supplemental data.docx [file IRNF_A_2578413_SM8973.docx]

Table S1.Baseline characteristics^[1]^

|  | SGLT2i at baseline (n = 877) | No SGLT2i at baseline (n = 12,149) |
| --- | --- | --- |
| Age, years | 61.8 ± 9.7 | 65.0 ± 9.5 |
| Sex |  |  |
| Male | 671 (76.5) | 8,417 (69.3) |
| Female | 206 (23.5) | 3,732 (30.7) |
| Race |  |  |
| White | 644 (73.4) | 8,225 (67.7) |
| Asian | 185 (21.1) | 2,709 (22.3) |
| Black/African American | 20 (2.3) | 502 (4.1) |
| SBP, mmHg | 133.3 ± 14.4 | 137.0 ± 14.2 |
| Duration of diabetes, years | 15.6 ± 8.1 | 15.4 ± 8.7 |
| HbA1c |  |  |
| % | 8.0 ± 1.2 | 7.7 ± 1.4 |
| mmol/mol | 63.5 ± 13.4 | 60.4 ± 14.9 |
| Serum potassium, mmol/L | 4.3 ± 0.4 | 4.4 ± 0.4 |
| eGFR, mL/min/1.73 m2 |  |  |
| Mean | 66.3 ± 21.1 | 57.0 ± 21.6 |
| Distribution |  |  |
| <25 | 0 | 162 (1.3) |
| 25 to <45 | 142 (16.2) | 4,090 (33.7) |
| 45 to <60 | 241 (27.5) | 3,193 (26.3) |
| ≥60 | 494 (56.3) | 4,701 (38.7) |
| UACR, mg/g |  |  |
| Median | 448 (185–945) | 521 (199–1,161) |
| Distribution |  |  |
| <30 | 16 (1.8) | 214 (1.8) |
| 30 to <300 | 283 (32.3) | 3,816 (31.4) |
| ≥300 | 578 (65.9) | 8,114 (66.8) |
| Medication use at baseline |  |  |
| RAS inhibitor | 875 (99.8) | 12,128 (99.8) |
| β-Blocker | 432 (49.3) | 6,072 (50.0) |
| Diuretic | 439 (50.1) | 6,271 (51.6) |
| Statin | 737 (84.0) | 8,662 (71.3) |
| Potassium supplement | 24 (2.7) | 361 (3.0) |
| Potassium-lowering agent | 7 (0.8) | 175 (1.4) |
| Glucose-lowering therapies |  |  |
| Insulin and analogs | 515 (58.7) | 7,115 (58.6) |
| Metformin | 692 (78.9) | 6,865 (56.5) |
| Sulfonylurea | 218 (24.9) | 3,171 (26.1) |
| DPP-4 inhibitor | 256 (29.2) | 3,022 (24.9) |
| GLP-1RA | 167 (19.0) | 777 (6.4) |
| α-Glucosidase inhibitor | 35 (4.0) | 621 (5.1) |
| Thiazolidinedione | 58 (6.6) | 459 (3.8) |

Data are mean ± SD, n (%), or median (interquartile range). DPP-4, dipeptidyl peptidase-4; eGFR, estimated glomerular filtration rate; GLP-1RA, glucagon-like peptide-1 receptor agonist; RAS, renin–angiotensin system; SBP, systolic blood pressure; SGLT2i, sodium–glucose cotransporter 2 inhibitor; UACR, urine albumin-to-creatinine ratio.

Table S2.Baseline distribution^[1]^

|  | SGLT2i at baseline | No SGLT2i at baseline |
| --- | --- | --- |
| CKD1/2 | 56.3% | 38.7% |
| CKD3 | 43.7% | 60.0% |
| CKD4 | 0.0% | 1.3% |

CKD, chronic kidney disease; SGLT2i, sodium-glucose cotransporter 2 inhibitors.

Table S3. Non-fatal CV events distribution^[1]^

| Event | Proportion |
| --- | --- |
| **Finerenone + SoC** |  |
| Non-fatal myocardial infarction | 32.26% |
| Non-fatal ischemic stroke | 33.03% |
| Non-fatal hemorrhagic stroke | 4.30% |
| Hospitalisation for heart failure | 30.41% |
| **SGLT2i + SoC** |  |
| Non-fatal myocardial infarction | 27.27% |
| Non-fatal ischemic stroke | 27.92% |
| Non-fatal hemorrhagic stroke | 3.64% |
| Hospitalisation for heart failure | 41.18% |
| **Finerenone + SGLT2i + SoC** |  |
| Non-fatal myocardial infarction | 34.73% |
| Non-fatal ischemic stroke | 35.56% |
| Non-fatal hemorrhagic stroke | 4.63% |
| Hospitalisation for heart failure | 25.08% |

SoC, standard of care; SGLT2i, sodium-glucose cotransporter 2 inhibitors.

Table S4. Cost-effectiveness results (4-year horizon).

|  | Cost(CNY) | incrCost(CNY) | QALYs | incrQALYs | NMB(CNY) |
| --- | --- | --- | --- | --- | --- |
| SGLT2i therapy | 70584.62 | 0.00 | 2.314 | 0 | 0.00 |
| Triple therapy | 62516.28 | -8068.34 | 2.347 | 0.034 | 17117.47 |
|  | Cost(CNY) | incrCost(CNY) | QALYs | incrQALYs | NMB(CNY) |
| Finerenone therapy | 77954.74 | 0.00 | 2.299 | 0 | 0.00 |
| Triple therapy | 62516.28 | -15438.46 | 2.347 | 0.048 | 28434.07 |

[1] ROSSING P, ANKER S D, FILIPPATOS G, et al. Finerenone in Patients With Chronic Kidney Disease and Type 2 Diabetes by Sodium–Glucose Cotransporter 2 Inhibitor Treatment: The FIDELITY Analysis [J]. Diabetes Care, 2022, 45(12): 2991-8.
